# Pitavastatin attenuates AGEs-induced mitophagy *via* inhibition of ROS generation in the mitochondria of cardiomyocytes

**DOI:** 10.7555/JBR.31.20160116

**Published:** 2018-04-16

**Authors:** Zhimin Zha, Junhong Wang, Shiling Li, Yan Guo

**Affiliations:** 1. Department of Gerontology, the First Affiliated Hospital of Nanjing Medical University, Nanjing, Jiangsu 210029, China; 2. Department of Cardiology, the First Affiliated Hospital of Nanjing Medical University, Nanjing, Jiangsu 210029, China; 3. Department of Cardioangiology, Shengze Hospital of Jiangsu Province, Suzhou, China.

**Keywords:** advanced glycation end products (AGEs), receptor for advanced glycation end products (RAGE), pitavastatin, autophagy, mitochondrial oxidation, oxidative stress

## Abstract

This study aimed to investigate whether pitavastatin protected against injury induced by advanced glycation end products products (AGEs) in neonatal rat cardiomyocytes, and to examine the underlying mechanisms. Cardiomyocytes of neonatal rats were incubated for 48 hours with AGEs (100μg/mL), receptor for advanced glycation end products (RAGE), antibody (1μg/mL) and pitavastatin (600 ng/mL). The levels of p62 and beclin1 were determined by Western blotting. Mitochondrial membrane potential (ΔΨm) and the generation of reactive oxygen species (ROS) were measured through the JC-1 and DCFH-DA. In the AGEs group, the expression of beclin1 was remarkably increased compared to the control group, while the expression of p62 was significantly decreased. AGEs also markedly decreased ΔΨm and significantly increased ROS compared with the control group. After treatment with RAGE antibody or pitavastatin, the level of beclin1 was markedly decreased compared with the AGEs group, but the level of p62 was remarkably increased. In the AGEs+ RAGE antibody group and AGEs+ pitavastatin group, ΔΨm was significantly increased and ROS was remarkably decreased compared with the AGEs group. In conclusion, AGEs-RAGE may induce autophagy of cardiomyocytes by generation of ROS and pitavastatin could protect against AGEs-induced injury against cardiomyocytes.

## Introduction

Advanced glycation end products (AGEs) are products of glycosylation reactions called the Maillard reaction, which continuously accumulate in many tissues or organs of humans and animals with the increase of age^[1^‒^2]^. AGEs play a role in the accelerated process of diabetic cardiovascular complications and atherosclerosis complications through interactions with the receptor for advanced glycation end products (RAGE)^[3^‒^4]^. Several studies have revealed that accumulation of AGEs can induce myocardial remodeling and cardiac diastolic dysfunction through oxidative stress^[5^‒^6]^. After binding to their receptors, RAGE, AGEs induce the generation of reactive oxidative species (ROS) that can lead to oxidative stress^[7]^.

Autophagy is a self-digestion process that delivers intracellular components to lysosomes through autophagosomes, which plays an important role in clearing the damaged macromolecular materials and cell organelles^[8]^. It is reported that autophagic cell death is induced in human umbilical vein endothelial cells treated with AGEs^[9]^. Oxidative stress and mitochondria damage are closely related to the occurrence of autophagy. It is well known that ROS induce autophagy^[10]^. So we speculate that oxidative stress, mitochondrial dysfunction, and autophagy may be associated with the AGEs-induced myocardial injury.

Statins, 3-hydroxy-3-methylglutaryl(HMG)-CoA reductase inhibitors, are now considered a first-line pharmacologic intervention for patients with dyslipidemia and atherosclerosis. The benefits of statins are well documented. However, myotoxic side effects including myopathy or rhabdomyolysis can sometimes be severe. Pitavastatin, a new statin agent, is associated with a low incidence of adverse metabolic events related to drug-drug interactions. In addition to lipid-lowering effect, pitavastatin plays an important role as an anti-atherosclerotic, anti-oxidant, anti-inflammatory agent and vascular endothelium protection^[11]^. In our current study, we investigated the effects of pitavastatin on AGE-induced cardiac autophagy, which will provide an experimental basis for its application to attenuate myocardial injury.

## Materials and methods

### Animals

Postnatal 1-3 day Sprague-Dawley rats were from the Experimental Animal Center of Nanjing Medical University. Hearts were harvested for analysis. All experimental procedures adhered to approval of the study by local committee and the National Institutes of Health guidelines on the use of experimental animals.

### Reagents

Pitavastatin was provided by Huawei Pharmaceutical Technology Development Co., (Fuzhou,China). AGE-modified bovine serum albumin (AGE-BSA) was purchased from Calbiochem (catolog number:121800, San Diego, USA). RAGE antibody was purchased from R&D (catolog number: AF1616, USA). Dulbecco's modified Eagle's medium (DMEM) and fetal bovine serum (FBS) were provided by Gibco (NY, USA). Collagenase II was purchased from Sigma (Santa Clara, USA). p62, beclin1, and GAPDH antibodies were obtained from CST (#5114, #3738, #2118, USA). Horseradish peroxidase (HRP)-conjugated goat anti-rabbit antibody was purchased from Abcam (6721, USA). Mitochondrial membrane potential assay kit with JC-1 and Reactive Oxygen Species assay kit were from Beyotime (Shanghai, China).

### Cell culture and treatment

Ventricular myocytes were obtained from 1 to 3 day old Sprague-Dawley rats. The hearts were removed quickly and washed three times with cold phosphate buffered saline (PBS). The left heart tissues were minced with scissors into 1 mm^3^ morsels and then digested with PBS containing 0.1% (w/v) collagenase II five to six times. Subsequently, the cell suspension was filtered with cell strainer and the mixture was centrifuged at 1,000 rpm for 10 minutes. The pellets were resuspended, transferred to culture plates, and incubated at 37°C. Fibroblasts were removed by adhesion onto the plates for 90 minutes. Non-adherent cells, neonatal rat ventricular cardiomyocytes, were plated in a culture plate and cultured in DMEM medium containing 10% (v/v) FBS, 100 U/mL penicillin, and 100μg/mL streptomycin under 5% CO_2_ atmosphere at 37°C. The medium was replaced every 48 hours. After 3 days' culture, when cells reached 80% confluence, cardiomyocytes were used for subsequent experiments.

Cardiomyocytes were divided into the following groups: (1) the control group; (2) the AGEs group that was treated with 100μg/mL AGE-BSA; (3) the AGEs+ RAGE antibody group that was pretreated with 1μg/mL RAGE antibody for 2 hours prior to a 48-hour exposure to AGE-BSA (100μg/mL); (4) the AGEs+ pitavastatin group that was pretreated with 600 ng/mL pitavastatin^[12]^ for 2 hours and then cocultured with AGE; and (5) the Pit group that was treated with 600ng/ml pitavastatin. After drug administration, cardiomyocytes were incubated for 48 hours.

### Total protein extraction

Cells were collected and lysed with RIPA Lysis Buffer (Beyotime) on ice, and then samples were centrifuged at 12,000 rpm for 20 minutes at 4°C. Supernatants were transferred to a new tube and the total protein concentrations were determined by a BCA Protein Assay Kit (Sigma, USA). After extraction, the protein samples were respectively stored at −20°C for further use.

### Mitochondrial membrane potential (ΔΨm) assay

Cardiomyocytes were incubated with JC-1 solution for 20 minutes at 37°C.Then cells were washed with JC-1 buffer for twice and medium was added to every well. Images were collected using a fluorescence microscope, which detects J-aggregates (excitation/emission= 525/590 nm) and JC-1 monomers (excitation/emission= 490/530 nm).

### Measurement of ROS

Intracellular ROS levels were assessed using DCFH-DA probe. Briefly, Cardiomyocytes were treated with 1ml DCFH-DA (10μmol/mL) for 20 minutes at 37°C. After incubation, the cells were washed with DMEM. The relative intensity of DCF fluorescence was determined at an ex/em wavelength of 488/525 nm as compared to control group cells.

### Western blotting

Protein expression levels of p53, p16, p62, Beclin1 and GAPDH were determined by western blotting. After the protein samples were heat-denatured, equal amounts of protein were separated by 10% SDS-PAGE and transferred to PVDF membranes by electrophoresis. The membranes were then blocked with 5% skim milk for 2 hours and incubated with primary antibodies at 4°C overnight. Membranes were subsequently washed by TBST buffer and incubated with the horseradish peroxidase-labeled goat anti-rabbit secondary antibody for 1 hour at room temperature. After washing the membrane by TBST for three times, the protein levels were detected by chemiluminescence using an enhanced chemiluminescence (ECL) detection kit (Pierce, France). The level of GAPDH was used as an internal control.

### Cell counting kit (CCK)-8 assays

Ventricular myocytes cells (100μl/well) were seeded into 96-well plate at a density of 10 × 10^4^ cells/ml and incubated at 37°C, 5% CO_2_ for 3 days. The cells were then treated with Pit of various concentration (0, 100, 200, 400, 600, 800, 1,000 and 1,200 ng/mL) for 48 hours. Following Pit treatment, a CCK-8 assay kit (Dojindo, Kumamoto, Japan) was used to detect cell viabilities according to the manufacturer's instructions. In brief, 10μL CCK-8 solution was added to each well. After 1‒4 hours incubation, cell viability was determined by measuring the absorbance at 450 nm using an ELISA reader (BioTek, Winooski, VT, USA).

### Statistical analysis

All data were obtained from at least 3 individual experiments. The results were presented as the mean±SEM and further analyzed by SPSS (ver.13.0). Differences between groups were determined using one-way ANOVA with the LSD multiple-comparison test when appropriate. Prism software (GraphPad) was adopted for statistical analysis. A final value of *P*≤0.05 was considered significant for all analyses.

## Results

### Pitavastatin attenuates AGEs-induced ROS production in cardiomyocytes

**Fig.1:**
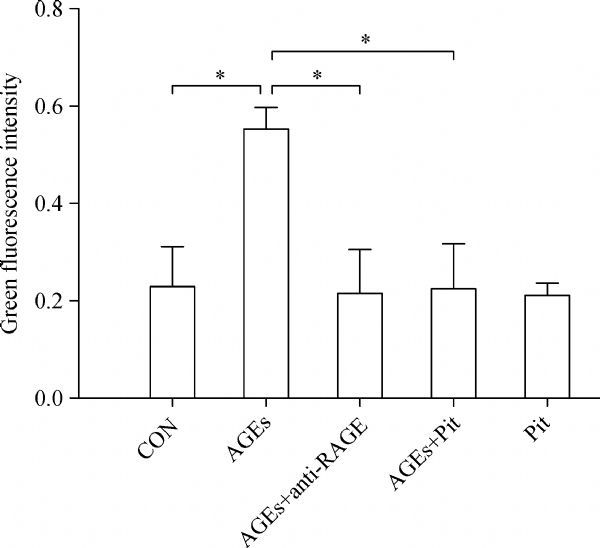
The effect of pitavastatin on AGEs-induced ROS. Intracellular ROS levels were assessed using DCFH-DA probe. Intensity alterations in ROS generation. Data represented are mean±SD of three independent experiments. *P < 0.01. AGE: advanced glycation end products; ROS: reactive oxygen species; Pit: pitavastatin.

Intracellular ROS levels were assessed using DCFH-DA probe. Our data showed that ROS levels in the AGEs-treated group were significantly higher than that in the control group (*P*<0.01; ***Fig. 1***). Pitavastatin or RAGE the antibody pretreatment resulted in significant attenuation of ROS formation in comparartion to AGEs group (*P*<0.01; ***Fig. 1***).

### Pitavastatin reduces AGEs-induced loss of mitochondrial membrane potential (ΔΨm)

**Fig.2:**
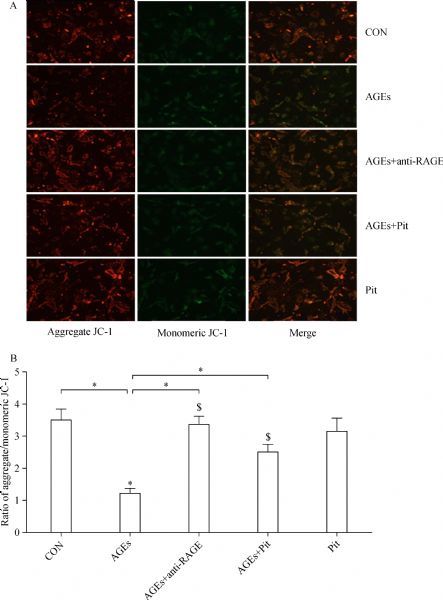
The effect of pitavastatin on AGEs-induced mitochondrial membrane potential (ΔΨm). Mitochondrial membrane potential was evaluated using JC-1 dye. Green fluorescence indicates low mitochondrial membrane potential, and red fluorescence indicates high mitochondrial membrane potential. (A) Fluorescence microscopy and (B) quantitative measurements of the fluorescence intensity uncovered alteration of intracellular mitochondrial membrane potential. Red-orange fluorescence came from JC-1 aggregates and green fluorescence came from JC-1 monomers. ΔΨm was assessed by JC-1 aggregates/JC-1 monomers ratios. Data are presented as mean±SD (n = 3 in each group). *P < 0.01; n = 3 in each group.

ΔΨm was monitored by using mitochondria-specific probe JC-1. In the mitochondria of healthy cells, JC-1 forms aggregates, which emit red fluorescence. However, it remains as monomers that emit green fluorescence during the loss of ΔΨm. The intensity ratio of red to green fluorescence represents the change in ΔΨm. As shown in ***Fig. 2A***, control cells showed mainly an intense red fluorescence coming from JC-1 aggregates. In contrast, two days of treatment with AGEs dramatically decreased ΔΨm of rat cardiomyocytes compared with control (*P*<0.01, ***Fig. 2***). However, combination of AGEs and pitavastatin or RAGE antibody attenuated AGEs-induced loss of mitochondrial ΔΨm in cardiomyocytes (*P*<0.01, ***Fig. 2***).

### Pitavastatin suppresses AGEs-induced autophagy in cardiomyocytes

**Fig.3:**
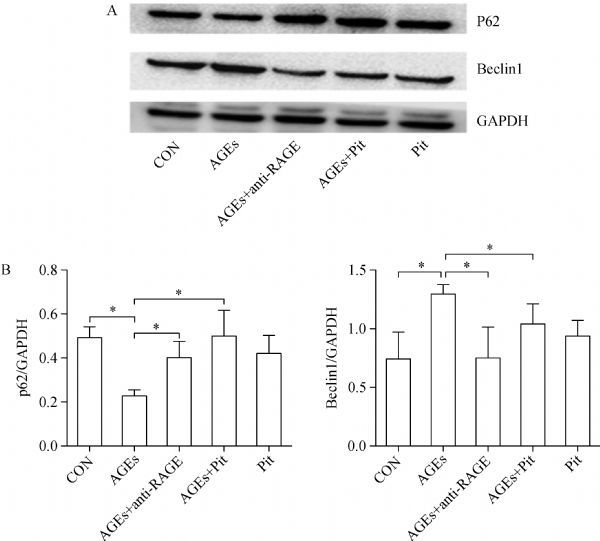
The effect of pitavastatin on AGEs-induced autophagy. A: Western blot analysis of Beclin1 and p62 protein levels. B: Protein quantification by densitometry. Data are presented as mean±SD (n = 3 in each group). *P < 0.05.

To determine the effects of pitavastatin on mitophagy in cardiomyocytes, we examined the expression of beclin1 and p62 by Western blotting. As shown in ***Fig. 3***, the expression of beclin1 was significantly increased, whereas p62 was decreased in AGEs-treated cells (*P*<0.05). The expression of p62 significantly increased after pretreatment with pitavastatin or RAGE antibody. However, the expression of beclin1 was decreased when the cells were adminstered with pitavastatin or RAGE antibody (*P*<0.05).

### Myocardial toxicity with increasing concentrations of pitavastatin

**Fig.4:**
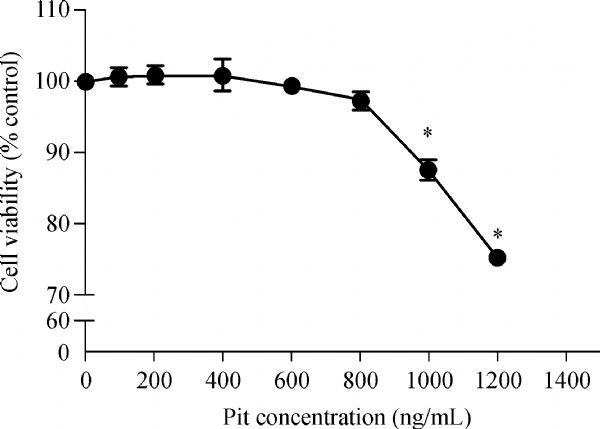
Effect of pitavastatin on rat myocardial cell activity. Data are presented as mean±SD (n = 3 in each group). *P < 0.05, versus the control group.

Myocardial cells were inoculated on 96-well plates with 1 × 10^4^ cells per well. After 72 hours, cells were treated with pitavastatin at different concentrations (0, 100, 200, 400, 600, 800, 1,000 and 1,200 ng/mL). The cell's activity was measured after culturing for 48 hours and the results are shown in ***Fig. 4***. CCK8 assay showed that low concentration of pitavastatin (100-800 ng/mL) had no significant influence on cell viability. However, when the Pit concentration increased to 1,000 and 1,200 ng/mL, cell toxicity was evident. Therefore, the concentration of 600 ng/mL in our experiment is appropriate.

## Discussion

Advanced glycation end products (AGEs) play an important role in the development of diabetic cardiomyopathy^[13]^. However, the underlying mechanisms are not completely understood. Oxidative stress plays a critical role in the development of structural and biochemical alterations of the heart^[14^‒^16]^. The detrimental effects of higher oxidative stress on cardiomyocytes include endothelial dysfunction, lipid peroxid-ation, apoptosis, activation of several inflammatory pathways etc^[17^‒^19]^. Accordingly, the inhibition of oxidative stress, an balance between formation and decomposition of ROS, is of great importance for cardiac injury. Currently, statins, in addition to their cholesterol lowering characteristics, are commonly prescribed for its antioxidant effects^[20^‒^21]^. In human umbilical vein endothelial cells, statins can decrease oxidized lowdensity lipoprotein (ox-LDL) and inhibit oxidative stress and cytotoxicity *via* decreasing ROS formation and downregulating NOX activity^[22^‒^23]^. In our study, we found evident increase of ROS in the AGEs group and remarkable downregulation after treatment with anti-RAGE antibody or pitavastatin, which indicates that pitavastatin may inhibit cardiac injury *via* downregulation of oxidative stress.

Mitochondrial membrane potential (ΔΨm) is necessary for production of energy and preservation of cellular homeostasis, which is a critical primary determinant of myocyte survival. In our study, we found that ΔΨm was downregulated after cultured cardiomyocytes were treated with AGEs. These demonstrated that AGEs can cause mitochondrial damage in cardiomyocytes. As ROS accumulation was a result of mitochondrial impairment, increased ROS, in turn, further can increase the loss of ΔΨ in the mitochondria of cardiomyocytes^[24]^, and further attenuates the bioenergetic function of mitochondria. This causes a vicious cycle that progressively aggravates oxidative stress and mitochondrial dysfunction in tissues and organs. Our data further demonstrated that two hours of pretreatment with pitavastatin significantly attenuated the loss of ΔΨm in mitochondria of cardiomyocytes, which may imply that pitavastatin can at least partially protect the cardiomyocytes by decreasing ROS generation and preserving the integrity of mitochondrial membrane.

Autophagy is a crucial process for the removal of misfolded proteins, damaged organelles and intracellular pathogens^[25]^. Autophagic flux is often assessed using p62 and beclin1 Western blotting. In our study, we found that the level of beclin1 was upregulated and p62 was downregulated after cultured cardiomyocytes were treated with AGEs. Similarly, a recent research indicates that AGEs triggers autophagy *via* the PI3K/AKT/mTOR signaling pathway in cardiomyocytes^[26]^. These demonstrated that autophagic flux was enhanced by AGEs in cardiomyocytes. Nevertheless, studies have yielded conflicting results. Some experiments showed that AGEs induce autophagic inactivation in vascular smooth muscle cell or under other conditions^[27^‒^28]^. It is as though autophagy is a double-edged sword in cardiomyocyte demise and survival. However, some evidences also suggest that autophagy, in addition to its role in cell survival, can also lead to cell death^[29^‒^31]^. Patschan *et al*. previously reported that autophagy induced by GC, an important component of AGEs, contributes to cell death in HUVECs^[32^‒^33]^. It is implied that autophagy may promote cell death through excessive self-digestion and degradation of essential cellular constituents. Intreastingly, pitavastatin stimulated the expression of p62 and inhibited the level of Beclin1 in our study. This result indicates that the mechanisms by which pitavastatin reversed the effect of AGEs appear to involve the autophagy pathway. In previous studies, statin therapy influences autophagic events. Statins like fluvastatin, potently affect macrophage viability *in vitro* through the induction of apoptotic process^[34]^. It is plausible that the early autophagic flux induced by statin treatment may be a potential mechanism in myocardial injury process, thus explaining in part the achieved beneficial effects.

In summary, our work proved that pitavastatin effectively inhibited AGEs-induced autophagic changes in cardiomyocytes of rats, and may alleviate myocardial injury induced by AGEs *via* inhibiting oxidative stress, protecting mitochondria and downregulating autophagy. Taken together, these results provide the rationale for possible therapeutic strageties for the treatment of diabetic cardiomyopathy.
